# 64180 The Proportion of Young Patients with Acute Surgical Pain and Development of Opiate Abuse Disorders

**DOI:** 10.1017/cts.2021.503

**Published:** 2021-03-30

**Authors:** Armando Uribe-Rivera, Linda Rasubala, Daniela Alvarez, Daniel Monroy-Giamundo, Hans Malmstrom, Yan-Fang Ren

**Affiliations:** University of Rochester Eastman Institute for Oral Health

## Abstract

ABSTRACT IMPACT: The importance of this study is to evaluate the responses following the exposure of opioid drugs in young adults to address the current opioid epidemics OBJECTIVES/GOALS: To compare the proportion of patients between 18 and 25 years of age, who develop an opioid abuse disorder following dental surgery, to those following other surgical procedures, when an opioid drug is used for acute postoperative pain control. METHODS/STUDY POPULATION: We fashioned an IRB-sponsored retrospective cohort study of patients, ages 18 to 25 years old, who presented for dental surgery or other medical surgical procedures, at Strong Memorial Hospital Medical Center, at the University of Rochester and received opioid drug treatments, for acute surgical pain management. Patients with the diagnosis of acute surgical pain were included in the study. However, those with chronic pain or other related abnormalities were excluded, even if a diagnosis of acute surgical pain was present in the electronic chart. The clinical data were retrieved from electronic medical records and NYS-iStop records. All statistics were significant at the level of <0.1 RESULTS/ANTICIPATED RESULTS: We identified 167 subjects, of whom, only 150 subjects met inclusion criteria (n=100) in dentistry and (n=50) in other medical specialties. Patients were followed up in a 7-year period. Most of the subjects were females (n=91), Caucasian (n=80), and lived in a suburban location (n=78). The most frequently prescribed opioid included hydrocodone (n=119). A significantly higher proportion (8.7%) of patients developed opioid abuse disorders in the control group, compared to 1% of subjects in the experimental group (p 0.02). Those in the control group received marginally significant higher doses of MMEs 447.2 +/-644.8 mg vs 306.2 +/-354.7mg in the experimental group (p 0.086). Those in the control group had significantly longer periods of opioid treatment 10 (+/-12) compared to 6(+/-6) days in the experimental group DISCUSSION/SIGNIFICANCE OF FINDINGS: It is paramount to evaluate the morphine milligram equivalents, and duration of opioid treatment given to the young population with acute surgical pain, to prevent opioid abuse disorders in this vulnerable cohort.

